# Knockdown of the T-box transcription factor Brachyury increases sensitivity of adenoid cystic carcinoma cells to chemotherapy and radiation *in vitro:* Implications for a new therapeutic principle

**DOI:** 10.3892/ijo.2014.2292

**Published:** 2014-02-06

**Authors:** YOSUKE KOBAYASHI, TSUYOSHI SUGIURA, IKUMI IMAJYO, MIYUKI SHIMODA, KOTARO ISHII, NAONARI AKIMOTO, NAOYA YOSHIHAMA, YOSHIHIDE MORI

**Affiliations:** 1 Sections of Oral and Maxillofacial Surgery, Kyushu University, Higashi-ku, Fukuoka 812-8582, Japan; 2 Oral and Maxillofacial Oncology, Division of Maxillofacial Diagnostic and Surgical Sciences, Faculty of Dental Science, Kyushu University, Higashi-ku, Fukuoka 812-8582, Japan

**Keywords:** adenoid cystic carcinoma cells, Brachyury, chemotherapy, radiation, cancer stem cell

## Abstract

Adenoid cystic carcinoma (AdCC) is highly metastatic and resistant to chemotherapy and radiotherapy. Recently, we reported that the T-box transcription factor Brachyury is a potential regulator of cancer stem cells (CSCs). Specifically, growth of CSCs was found to be controlled by *Brachyury* knockdown in AdCC. Since CSCs are resistant to chemotherapy and radiotherapy, this finding provides a new principle for therapies targeting CSCs. In the present study, we established that *Brachyury* knockdown suppresses chemoresistance and radioresistance *in vitro. Brachyury* was knocked down by transfecting Brachyury short hairpin RNA (shRNA) into the AdCC CSC cell line ACCS-M GFP. *Brachyury* knockdown significantly inhibited cell migration and invasion and suppressed chemoresistance. A quantitative PCR array of drug transporter genes revealed that knockdown of *Brachyury* caused downregulation of ATP-binding cassette transporter genes. Furthermore, ACCS-M GFP radioresistance was significantly suppressed by *Brachyury* knockdown. Knockdown of *Brachyury* significantly sensitized ACCS-M GFP cells to chemoradiotherapy. This study demonstrates that *Brachyury* knockdown reduces invasiveness and chemoresistance and radioresistance of CSCs *in vivo.* Therefore, *Brachyury* knockdown may be a useful therapeutic tool for sensitizing CSCs to conventional chemoradiotherapy.

## Introduction

Adenoid cystic carcinoma (AdCC) is among the most common malignant tumors of the salivary glands and is characterized by unique clinical features and behavior. Although slow growing, AdCC spreads relentlessly into adjacent tissues. It carries a high risk of recurrence and distant metastases, with 40–60% of afflicted patients developing distant metastases to the lungs, bone, and soft tissues (1,2). AdCC is resistant to chemotherapy and radiotherapy. Therefore, treatment-resistant distant metastases remain a significant obstacle to the long-term cure of patients with AdCC, emphasizing the need for anti-metastasis therapy for AdCC.

We previously established 3 AdCC cell lines that express green fluorescent protein (GFP) from the ACCS cell line using orthotopic transplantation and *in vivo* selection in the nude mouse. These 3 lines include the parental ACCS GFP, the highly tumorigenic ACCS-T GFP, and the metastatic ACCS-M GFP line. We demonstrated that ACCS-M GFP cells exhibited a loss of E-cadherin and integrins and a gain in vimentin, suggesting that the epithelial-mesenchymal transition (EMT) is a key event in AdCC metastasis that induces tumor cell dissemination from the primary tumor site (3). We also showed a direct correlation between EMT and prevalence of cancer stem cell-like cells in AdCC (4).

The EMT program triggered during tumor progression appears to be controlled by expression of early embryonic genes, including *Twist, Snail, Slug, Goosecoid* and *SIP1* (5,6). The transcription factors encoded by these genes impart mesenchymal traits to tumor cells, including motility and invasiveness. For example, expression of *Twist* is elevated in various cancers, including breast, prostate, gastric and melanoma (7). In addition, the T-box transcription factor Brachyury, a protein required for mesoderm formation during development (8–10), reportedly promotes EMT in human carcinoma cell lines (11). The latter study also showed that *Brachyury* overexpression in human carcinoma cells induced changes characteristic of EMT. These findings suggest that the EMT in cancer cells is controlled by mechanisms similar to the EMT during normal human development.

Other studies using neoplastic tissue have identified self-renewing, stem-like cells within tumors, referred to as cancer stem cells (CSCs). CSCs constitute a minority of neoplastic cells within a tumor and are defined operationally by their ability to seed new tumors. For this reason, they have also been termed tumor-initiating cells (12). During the process of tumor metastasis, which is often enabled by EMT (13), disseminated cancer cells are thought to require self-renewal properties similar to those exhibited by stem cells in order to spawn macroscopic metastases. This raises the possibility that the EMT process, which enables cancer cell dissemination, may also impart self-renewal to disseminating cancer cells. Indeed, emerging evidence of a direct interaction between EMT and CSCs (11,14,15). Similarly to normal stem cells, CSCs are regulated by key genes, such as *Oct4, Nanog, c-Myc, Sox2,* and *Klf4,* which are similar to EMT-regulator genes (16,17). CSCs are resistant to chemotherapy and radiotherapy (18,19), suggesting a new therapeutic principle for targeting CSCs (20,21).

We have confirmed a direct interaction between the EMT and CSCs in the highly metastatic AdCC subclone ACCS-M GFP. We also reported that the T-box transcription factor Brachyury, which is also a marker of mesoderm differentiation (22,23), regulates CSC and the EMT in AdCC cells. *Brachyury* knockdown exerted a stronger effect on cancer sternness and EMT phenotype than did knockdown of the conventional CSC regulator gene, *Sox2.* By reducing the sternness of CSCs, *Brachyury* knockdown significantly inhibited tumorigenicity and metastasis *in vivo* (4). This hypothesis has been supported by recent evidence linking Brachyury to CSCs in colon cancer (24).

These observations suggest that knocking down *Brachyury* can control CSC and EMT, thus inducing CSC differentiation and sensitization to conventional chemotherapy and radiotherapy. In this study, we validated that *Brachyury* knockdown suppresses chemo- and radioresistance *in vitro* as a first step in establishing its therapeutic potential against CSCs.

## Materials and methods

### Chemicals

Standard anticancer drug kits were provided by Scientific Support Programs for Cancer Research, Grant-in-Aid for Scientific Research on Innovative Areas from the Ministry of Education, Culture, Sports, Science and Technology of Japan. Docetaxel, 5-fluorouracil (5-FU), pacli-taxel, cisplatin (CDDP), mitomycin C, bestatin hydrochloride, bleomycin sulfate and etoposide were purchased from Sigma-Aldrich (St. Louis, MO, USA). Actinomycin D and streptomycin-SP were purchased from Calbiochem (Merck, Darmstadt, Germany).

### Cells and cell culture

The human cell lines ACCS, ACCS GFP and ACCS-M GFP were established in our laboratory as previously described (3). Briefly, the parental cell line ACCS and the GFP-transfected sub-line ACCS GFP displayed similar morphology, growth rate and tumorigenicity *in vitro* and *in vivo.* Similar to the parental ACCS cells, ACCS GFP cells had low tumorigenicity (22.2% incidence). Using ACCS GFP cells injected into the tongue of nude mice, tumor formation was observed under the excitation wavelength. Green fluorescence was not observed in the absence of tumors. We performed *in vivo* selection of clones with higher tumorigenicity by repeatedly recovering cells *in vitro* and transplanting them into the tongue of nude mice. This selection process yielded a subline exhibiting high tumorigenicity (100% incidence) and high frequency of metastasis to submandibular lymph nodes (100% incidence); these cells were termed ACCS-M GFP. The histological and immunohistochemical features of ACCS-M GFP tumors were similar to the solid pattern of AdCC. The cell lines were maintained as a monolayer culture in Dulbecco’s modified Eagle’s medium (DMEM; Sigma-Aldrich) supplemented with 10% fetal bovine serum (FBS; ICN Biomedicals, Aurora, OH, USA), 2 mM 1-glutamine, penicillin G, and streptomycin in a humidified incubator under an atmosphere of 5% CO_2_ at 37°C (3).

### Transfection of Brachyury and SOX2 shRNA

Cultured ACCS cells were transfected with short hairpin RNA (shRNA) lentiviral plasmids (pLKO.l-puro; Sigma-Aldrich) using Lipofectamine LTX (Invitrogen Life Technologies, Carlsbad, CA, USA) according to the manufacturer’s instructions as previously described (4). ACCS-M sh cont. cells were generated by transfecting ACCS-M GFP cells with pLKO.l-puro Control shRNA Vector (Sigma-Aldrich). ACCS-M shBr and ACCS-M shSOX2 cells were generated by transfecting ACCS GFP and ACCS-M GFP cells with pLKO.l-puro/sh. Brachyury or pLKO.l-puro/sh. SOX2 (Sigma-Aldrich), respectively. Colonies resistant to puromycin (Sigma) were pooled from the individual transfection experiments. The expression level of *Brachyury* in shRNA-transfected ACCS cells was monitored by real-time reverse transcription-PCR (RT-PCR) (4). All transfected cells were maintained in DMEM containing 10% FBS and 2*μ*g/ml puromycin (Sigma-Aldrich).

### Wound healing assay

Cells (3×l0^5^) were seeded on a 6-well plate. After 24 h, ‘wounds’ were scratched with a 200-*μ*l pipette tip, washed with medium and observed under a fluorescence microscope (BZ-8000; Keyence, Osaka, Japan). The wound regions were photographed again after 8, 16 and 24 h, and the wound areas were measured. Wound area was calculated using the following formula: wound area (% of control) = (wound area after the indicated period × l00)/initial wound area.

### Evaluation of tumor dissemination from the primary cancer nest

Evaluation of tumor dissemination from the primary cancer nest was performed as previously described (25). Briefly, living ACCS cell lines were fluorescently labeled using Vybrant DiO and DiD cell-labeling solutions (Molecular Probes, Eugene, OR, USA) according to the manufacturer’s instructions. Then, l×l0^6^ labeled cells were pelleted and resuspended in 10 *μ*l collagen type I gel to form a solid cell cluster. The collagen-embedded tumor cell pellets were allowed to solidify for 30 min at 37°C in a 100-*μ*l microcentrifuge tube; the pellets were then embedded in non-labeled fibroblasts containing collagen type I gel (1×10^5^ cells/ml) and solidified. Growth medium was placed over the collagen gels and cultured. Tumor dissemination was observed under a fluorescence microscope (BZ-8000; Keyence). The grade of tumor dissemination from the tumor cell pellet (modeling the primary tumor nest) was evaluated by measuring the distance of all cells from the edge of the nest in 5 randomly selected, standardized rectangular light fields (500×100 *μ*m), and the values were summed. The evaluation was conducted twice daily for 7 days.

### MTT assay

ACCS cell lines were seeded into CellTiter 96 Aqueous Non-radioactive Cell Proliferation Assay G4000 plates (Promega, Madison, WI, USA) at a density of 5×l0^3^ cells per well and incubated in DMEM containing 10% FBS for 8 h. The medium was replaced with serum-free DMEM after 3 washes with PBS. For chemosensitivity analysis, a dilutional series of anticancer drugs was applied at final drug concentrations of 0, 0.001, 0.01, 0.1, 1, 10, 100 and 1,000 *μ*M and incubated for 24 h in a humidified incubator under an atmosphere of 5% CO_2_ at 37°C. For radiosensitivity analysis, *in vitro* gamma-ray irradiation was administered at 5,10,15, or 30 Gy with a Gammacell 40^®^ Exactor Low Dose-Rate Research Irradiator (Best Theratronics, Ottawa, Canada), and cells were then incubated for 48 or 72 h in a humidified incubator under an atmosphere of 5% CO_2_ at 37°C. After incubation, ACCS cells were analyzed by CellTiter 96 Aqueous Non-radioactive Cell Proliferation Assay G4000 (Promega) according to the manufacturer’s instructions. The absorbance of samples at 590 nm (A590) was measured with a microplate reader (Model 680, Bio-Rad, USA). All experiments were carried out in triplicate and repeated 3 times.

Data were normalized to the untreated controls and reported as % viability. The IC_50_ (*μ*M) values for cytotoxicity of the anticancer drug represents the concentration yielding 50% viability, which was determined from the concentration-viability curve. The concentration-viability curve was generated using a non-linear regression model with the Solver function of Microsoft Excel as previously described (26).

### Real-time RT-PCR

Total RNA was extracted from ACCS GFP cells using the RNeasy Mini kit (Qiagen, Chats worth, CA, USA) and used for first-strand cDNA synthesis. The mRNA levels were quantified in triplicate using a real-time PCR system with the Brilliant SYBR Green qPCR kit (Stratagene, La Jolla, CA, USA) for *Brachyury* and *Sox2* or the RT^2^ Profiler PCR Array (96-well format) for human drug transporters (Qiagen). Specific primers for *Brachyury* and *Sox2* were: hBrachyury (F) 5’-TGCTGCAATCCCATGACA-3’, (R) 5’-CGTTGCTCACAGACCACA-3’; hSOX2, (F) 5’-TGG GTTCGGTGGTCAAGT-3’, (R) 5’-CTCTGGTAGTGCTG GGACA-3’. The PCR cycling conditions were 10 min at 95°C followed by 47 cycles at 95°C for 30 sec, 60°C for 30 sec, and 72°C for 60 sec. Dissociation curve analyses confirmed that the signals corresponded to unique amplicons. Expression levels were normalized to (β-actin mRNA levels for each sample obtained from parallel assays and analyzed using the LightCycler software package version 3.5 (Roche Diagnostics, Mannheim, Germany) for hBrachyury and hSOX2 and Mx 3000P QPCR system (Agilent Technologies, CO, USA) for the RT^2^ Profiler PCR Array.

### Statistical analysis

All data are represented as mean ± SD, as analyzed via analysis of variance and Student’s t-test, and processed using the statistical software SPSS 13.0. Statistical significance was defined as P<0.05.

## Results

### Brachyury and SOX2 shRNA do not influence growth of ACCS cell lines

**Figure 1.**
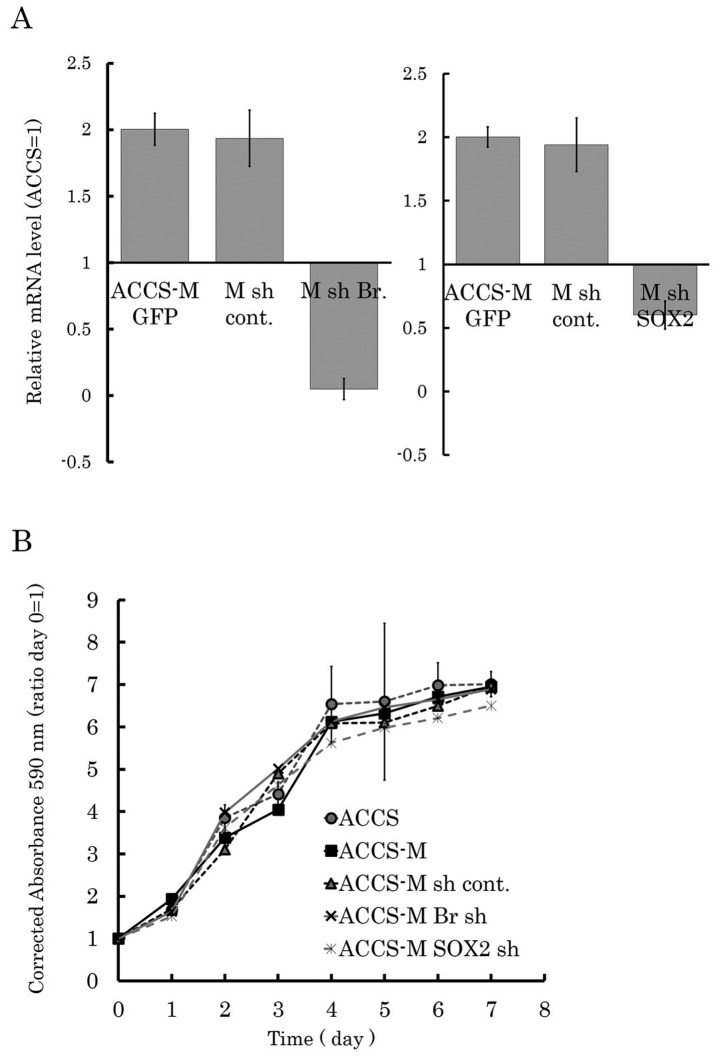
Effect of *Brachyury* and *SOX2* knockdown on ACCS cell growth. (A) The mRNA expression levels of *Brachyury* and *Sox2* in shRNA-transfected cells as quantified by real-time RT-PCR. Data are shown as mRNA levels relative to ACCS GFP. Error bars indicate the standard deviation. (B) Cell growth of ACCS and its derivatives, as evaluated by MTT assay. Error bars indicate the standard deviation.

We established ACCS GFP and ACCS-M GFP-derived cell lines by stable transfection of *Brachyury* or *SOX2* shRNA lentiviral plasmids. The expression level of *Brachyury* or *SOX2* in shRNA-transfected ACCS cells was monitored by real-time RT-PCR to confirm silencing of the target genes (Fig. 1A). We first analyzed the effect of *Brachyury* or *SOX2* knockdown on cell growth *in vitro* by MTT assay. Cancer stem cell-like ACCS-M GFP cells demonstrated a similar growth pattern to parental ACCS GFP cells. Stable transfection of shRNA did not affect cell growth (ACCS-M sh cont. GFP). Neither Brachyury shRNA nor SOX2 shRNA affected cell growth (ACCS-M shBr GFP and ACCS-M shSOX2 GFP, respectively; Fig. 1B).

### Brachyury shRNA inhibits cell migration

**Figure 2.**
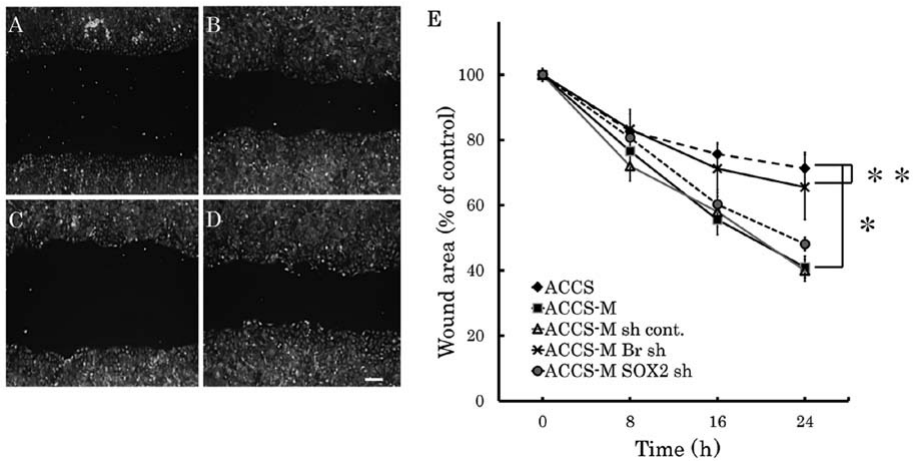
Effect of *Brachyury* and *SOX2* knockdown on ACCS cell migration. Cell migration, as evaluated by the wound-healing assay. Photomicrographs of wound regions 24 h after the start of the assay (A–D). ACCS GFP (A), ACCS-M GFP (B), ACCS-M shBr GFP (C), and ACCS-M shSOX2 GFP (D) cells. Bar, 100 *μ*m. (E) Calculated wound area. ^*^P<0.001, ^**^P<0.05.

The effect of *Brachyury* or *SOX2* knockdown on cell migration *in vitro* was analyzed by the wound healing assay (Fig. 2). Cell migration of ACCS-M GFP cells was approximately twice as fast as that of ACCS GFP cells. *Brachyury* knockdown significantly inhibited migration of ACCS-M GFP cells to the level of parental ACCS GFP (P=0.001). By contrast, *SOX2* knockdown had no effect on ACCS-M GFP cell migration.

### Brachyury and SOX2 shRNA inhibit cell invasion

**Figure 3.**
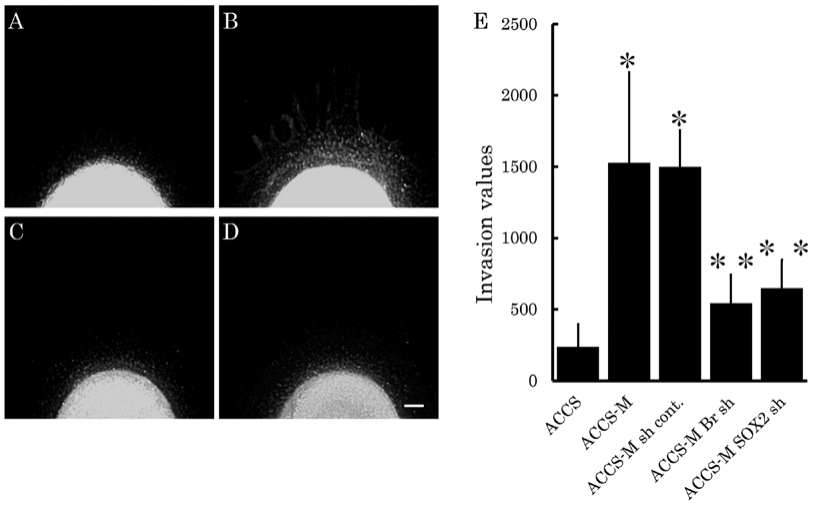
Effect of *Brachyury* and *SOX2* knockdown on ACCS cell invasiveness. ACCS GFP (A), ACCS-M GFP (B), ACCS-M shBr GFP (C), and ACCS-M shSOX2 GFP (D) cells as analyzed with the new invasion assay. Photomicrographs taken after a 7-day incubation. Bar, 100 *μ*m. (E) Error bars indicate standard deviation. ^*^P<0.001, ^**^P<0.05.

We next analyzed the effect of *Brachyury* or *SOX2* knockdown on cell invasiveness *in vitro* using our previously reported tumor-cell dissemination assay (25). In this assay, invasion of carcinoma cells is visualized as small green fluorescent spots escaping from a cell pellet that models the primary cancer nest. Therefore, we evaluated cancer cell invasion by the number of invasive cells and their distance from the artificial primary cancer nest. As shown in Fig. 3B, ACCS-M GFP cells demonstrated aggressive cell invasion into artificial stromal tissue. Invasiveness of ACCS-M GFP cells was strongly inhibited by knockdown of *Brachyury* (Fig. 3C) or SOX2 (Fig. 3D). Fig. 3E compares invasiveness among ACCS cell lines. Relative invasiveness values (ACCS GFP = 1) were 6.4 (ACCS-M GFP), 2.3 (ACCS-M shBr GFP), and 3.2 (ACCS-M shSOX2 GFP).

### Brachyury and SOX2 shRNA induce chemosensitivity in vitro

**Figure 4.**
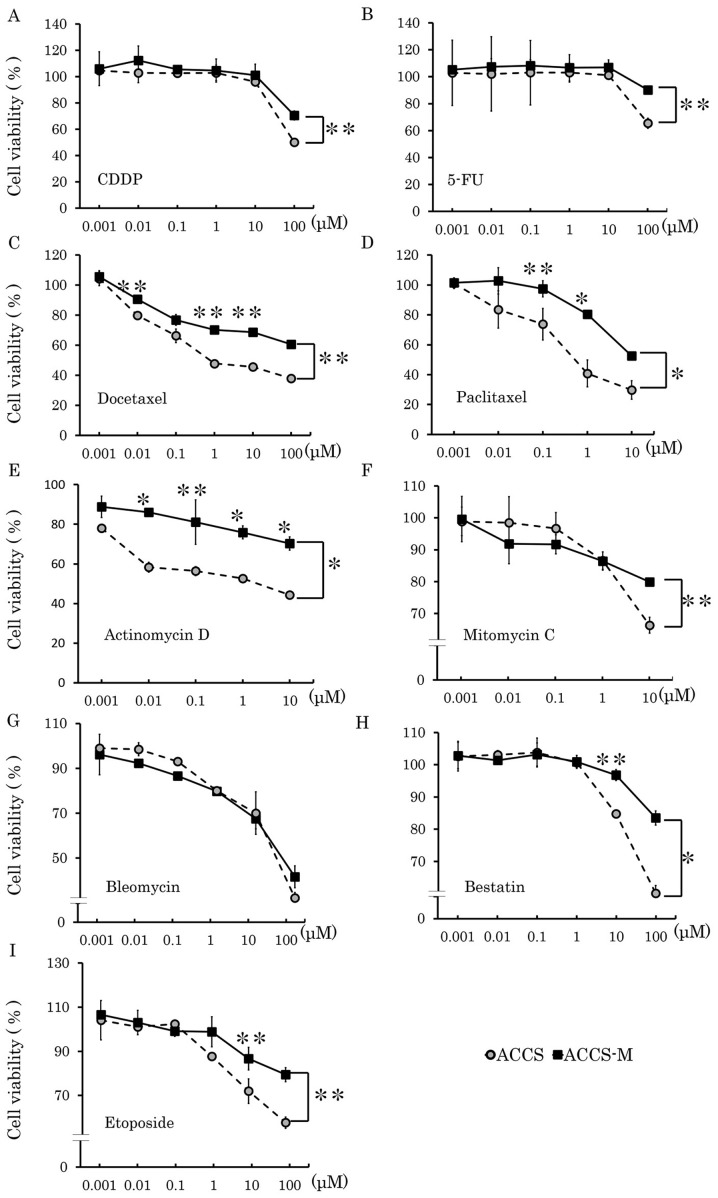
Comparison of chemosensitivity between ACCS GFP and ACCS-M GFP cells (A–l). Cell viability after application of anticancer drugs to ACCS GFP and ACCS-M GFP cells at final concentrations of 0,0.001,0.01,0.1,1,10, or 100 *μ*M. Error bars indicate standard deviation. ^*^P<0.001, ^**^P<0.05.

Cancer stem cells are known to resist various types of anticancer drugs. Therefore, we next assessed whether knockdown of cancer stem cell regulators could change their sensitivity to anticancer drugs. ACCS-M GFP cells demonstrated chemoresistance to CDDP, docetaxel, actinomycin D, etoposide, 5-FU, paclitaxel, mitomycin C and bestatin (Fig. 4). Table I shows the IC_50_ values of each anticancer drug in each ACCS cell line. The IC_50_ values of ACCS-M GFP cells were higher than those of ACCS GFP cells for each anticancer agent (range: 1.2–355-fold). In particular, resistance to taxane drugs, docetaxel and paclitaxel, was very high (355- and 23-fold of ACCS GFP IC_50_ values, respectively). Brachyury shRNA and SOX2 shRNA reduced chemoresistance of ACCS-M GFP cells, but the effect of Brachyury shRNA was greater than that of SOX2 shRNA (Fig. 1 and Table I). Relative IC_50_ values (ACCS-M GFP=1) of Brachyury shRNA were ∼0.33 (docetaxel) to 0.85 (mitomycin C and bleomycin), and those of SOX2 shRNA were 0.59 (bestatin) to 1 (paclitaxel, mytomycin C, and bleomycin). However, with the exception of bestatin, the degree of reduction did not reach parental ACCS GFP levels (Table I). We could not determine an IC_50_ for etoposide, because ACCS cell lines showed strong resistance, and the maximum dose of etoposide (1,000 *μ*M) did not reduce cell viability to 50%.

### Brachyury and SOX2 shRNA modify expression of drug-transporter genes in vitro

**Figure 6.**
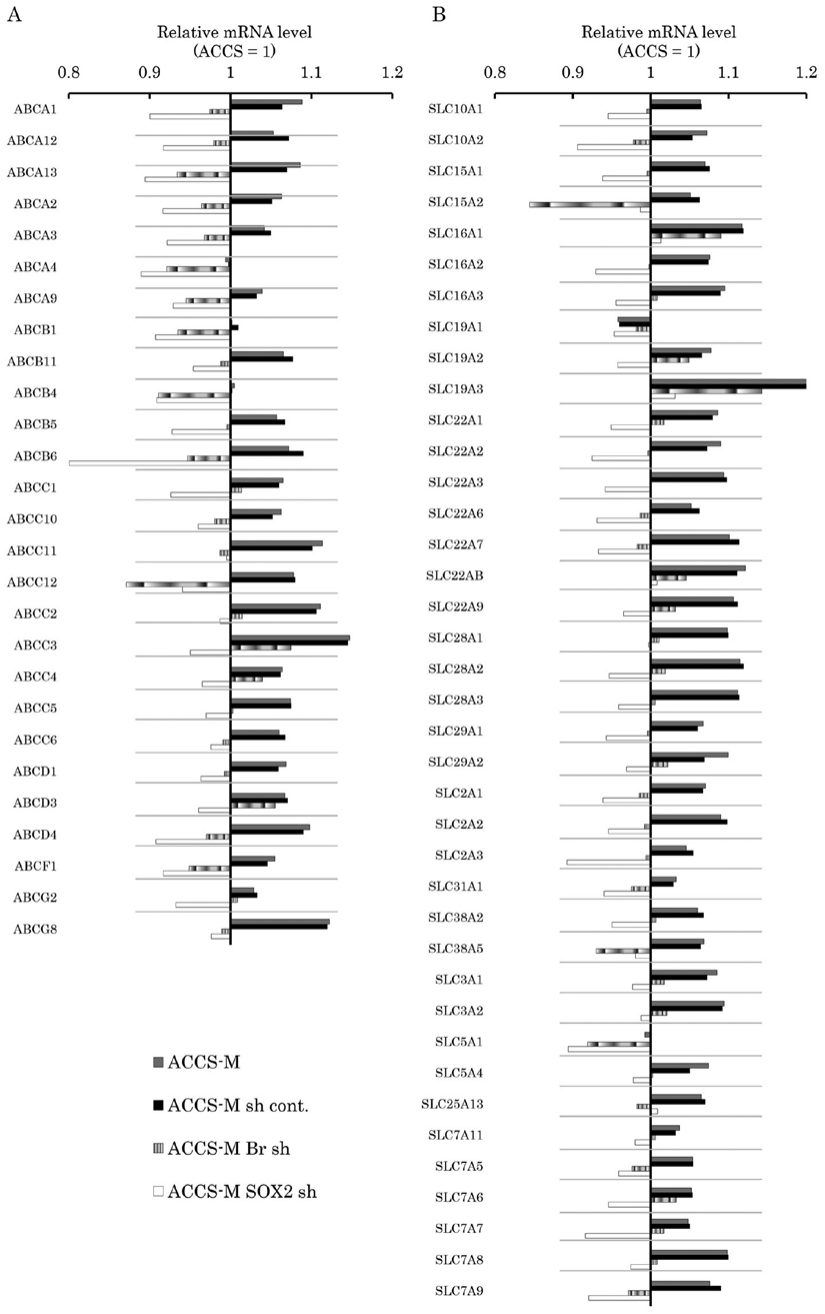
Effect of *Brachyury* and *SOX2* knockdown on mRNA expression of drug transporters. mRNA expression levels of the indicated genes in ACCS-M GFP cells and derivatives as quantitated by real-time RT-PCR. mRNA levels are reported as mRNA relative tp ACCS GFP. The expression levels of ABC transporter genes (A) and SLC family genes (B) are shown. Error bars indicate standard deviation.

Multidrug resistance in cancer is heavily dependent on 2 major super families of membrane transporter proteins that influence the pharmacokinetics of drugs, ATP-binding cassette (ABC) transporters and solute-carrier (SLC) transporters. Therefore, we analyzed the effect of *Brachyury* and *SOX2* knockdown on the expression levels of these membrane transporters by real-time PCR array. The differences between ACCS-M GFP and ACCS GFP in expression of these membrane transporters were not significant (1.05–1.2-fold). Notably, ACCS-M GFP cells generally expressed higher levels of ABC transporter genes than ACCS GFP cells, and this expression was reduced by *Brachyury* or *SOX2* knockdown (Fig. 6A). SLC transporter genes were also generally expressed at higher levels in ACCS-M GFP cells with the exception of *SLC19A1* and *SLC5A1. Brachyury* knockdown increased the expression of only one SLC transporter gene, *SLC19A1* (Fig. 6B).

### Brachyury and SOX2 shRNA induce radio sensitivity in vitro

**Figure 7.**
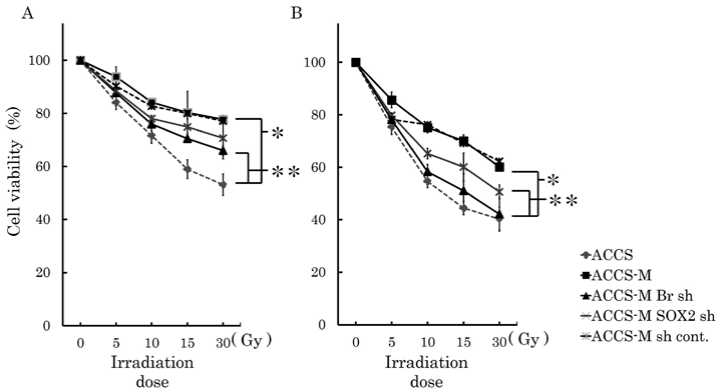
Effect of *Brachyury* and *SOX2* knockdown on radiosensitivity of ACCS-M GFP cells. Cell viability after various doses of radiation in ACCS GFP, ACCS-M GFP, ACCS-M sh cont. GFP, ACCS-M shBr GFP, and ACCS-M shSOX2 GFP cells after 48 h (A) and 72 h (B). Error bars indicate the standard deviation. ^**^P<0.05.

Cancer stem cells are insensitive to radiation. We analyzed sensitivity to radiation treatment *in vitro* and found that ACCS-M GFP cells were significantly more resistant to irradiation than ACCS GFP cells (P<0.001). The viability of ACCS GFP and ACCS-M GFP cells was 53 and 77%, respectively, 48 h after 30-Gy irradiation and 40 and 60% 72 h after 30-Gy irradiation, respectively. *Brachyury* and *SOX2* knockdown reduced cell viability 72 h after 30-Gy irradiation. *Brachyury* knockdown reduced cell viability upon irradiation significantly more than SOX2 knockdown (P<0.05) and increased the radiosensitivity of ACCS-M GFP to the level of ACCS GFP cells (Fig. 7).

### Brachyury and SOX2 shRNA enhance the cytotoxicity of concurrent chemoradiation treatment in vitro

**Figure 8.**
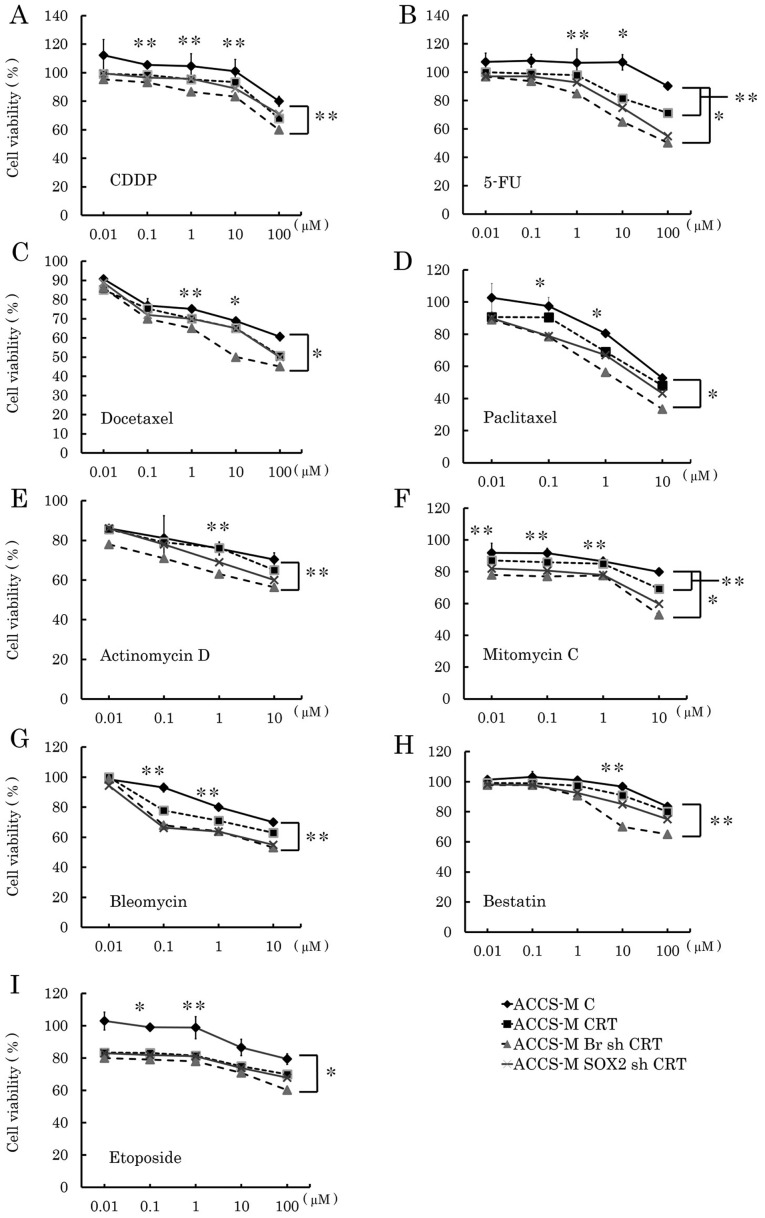
Effect of *Brachyury* and *SOX2* knockdown on chemoradiation treatment of ACCS-M GFP cells (A–I). Cell viability after radiation (30 Gy) and various types and concentrations of anticancer agents in ACCS-M GFP, GFP ACCS-M shBr GFP, and ACCS-M shSOX2 GFP cells. Values are relative to non-irradiated ACCS-M GFP cells. Error bars indicate standard deviation. ^*^P<0.001, ^**^P<0.05

We analyzed the effect of Brachyury and SOX2 shRNA on concurrent chemoradiation treatment (CRT) *in vitro* to assess the clinical potential of Brachyury and SOX2 shRNA. Fig. 8 compares anticancer drugs with or without radiation treatment. Brachyury and SOX2 shRNA demonstrated significantly affected CRT. Brachyury shRNA was more effective than SOX2 shRNA and significantly affected 5-FU, docetaxel, paclitaxel, mitomycin C, and etoposide CRT (P<0.001). Notably, Brachyury shRNA was also significantly effective for CRT with bleomycin and mitomycin C (both P<0.05), while Brachyury shRNA did not affect any single chemotherapy treatment.

## Discussion

Clinically, oral AdCC is resistant to chemotherapy and radiotherapy, which poses a major obstacle to treatment. CSCs may contribute to chemo- and radioresistance, and new cancer therapies targeting CSCs are under investigation (27–29). Recently, we reported that T-box transcription factor Brachyury is a putative factor underlying the EMT and CSC sternness of AdCC *in vitro* and metastasis *in vivo* and that *Brachyury* knockdown inhibits AdCC tumorigenicity and metastasis (4). Therefore, clinical application of *Brachyury* knockdown may be effective for inhibiting cancer metastasis. It remains to be determined whether *Brachyury* knockdown in pre-existing cancer reduces the invasiveness of CSCs in the primary nest and increases their sensitivity to chemo- and radiotherapy.

Cellular invasiveness and migration were markedly higher in ACCS-M GFP than in ACCS GFP cells. *Brachyury* knockdown completely inhibited cellular invasiveness and migration, while *SOX2* knockdown did not. Activation of cellular invasiveness is an important characteristic of EMT. Matrix metalloproteinases (MMPs) are upregulated in EMT and induce cellular invasion of cancer cells (30–32). Moreover, EMT-related MMP-9 upregulation degrades cell-surface E-cadherin (33), an important phenotype of EMT. Thus, *Brachyury* knockdown inhibited not only tumorigenicity and metastasis, but also cancer cell invasion at the primary site. This finding suggests that Brachyury knockdown can inhibit cancer cell invasiveness of pre-existing cancers.

CSCs are chemoresistant. Similarly, ACCS-M GFP cells demonstrated resistance to all tested anticancer drugs except bleomycin. The mechanism underlying chemoresistance involves drug transporters. Genetic variations in efflux transporters of the ABC family, such as *ABCB1* (MDR1, P-glycoprotein), *ABCC1* (MRP1), *ABCC2* (MRP2), and *ABCG2* (BCRP), and uptake transporters of the SLC family, such as *SLC19A1* (RFC1) and *SLC01B1* (SLC21A6), are implicated in resistance to chemotherapy (34,35). We found that nearly all ABC family genes were expressed at higher levels in ACCS-M GFP than in ACCS GFP cells, and this difference in expression may underlie the chemoresistance of ACCS-M GFP cells. However, the degree of upregulation of these genes was not large (∼5–15%), suggesting that another crucial factor underlies the chemoresistance of ACCS-M GFP.

*Brachyury* and *SOX2* knockdown inhibited the expression of ABC family genes. The *SOX2* gene enhances *ABCC3* and *ABCC6* expression through direct transcriptional regulation (36). *Brachyury* knockdown inhibited *SOX2* expression. Therefore, *Brachyury* may indirectly inhibit ABC transporter genes thorough *SOX2* downregulation. By contrast, SLC family genes were upregulated in ACCS-M GFP cells, suggesting that drug uptake into cancer cells is induced in ACCS-M GFP cells. This finding contradicts the observed chemoresistance of ACCS-M GFP. However, only *SLC19A1* (RFC1), the most relevant gene in chemoresistance (37,38), was decreased in ACCS-M GFP cells, which indicates that drug uptake was inhibited. Furthermore, *Brachyury* knockdown recovered expression of *SLC19A1* (RFC1) to its level in ACCS GFP cells, indicating that *Brachyury* knockdown induced drug uptake.

Another possible explanation for the drug resistance of CSCs is the characteristic proportion of CSCs at each stage of the cell cycle, because various anticancer drugs are cell cycle-dependent. CSCs have a significantly higher proportion of cells in the G2-phase of the cell cycle (39). Bleomycin, a glycopeptide antibiotic with a unique mechanism of antitumor activity, has G2-phase-specific cytotoxicity (40). Our results showed that only the cytotoxicity of bleomycin was unchanged by *Brachyury* knockdown in ACCS-M GFP cells. By contrast, ACCS-M GFP cells demonstrated resistance to the cell cycle-specific anticancer drugs 5-FU [S-phase (41)], etoposide [S/G2-phase (42)], and taxanes [docetaxel and paclitaxel, G2/M-phase (43)], which was reduced by *Brachyury* knockdown. These results suggest that CSC resistance to cell cycle-specific anticancer agents is partially regulated by G2-phase elongation in CSCs and that *Brachyury* knockdown can break the cell cycle arrest in CSCs.

Cancer stem-like cells are relatively radioresistant owing to intrinsic and extrinsic factors, including quiescence, radiation-response mechanisms (e.g., enhanced DNA repair, upregulated cell cycle-control mechanisms, and increased free-radical scavengers), and a microenvironment that enhances cell survival mechanisms (e.g., hypoxia and interaction with stromal elements) (44). Therefore, the same mechanisms of cell cycle regulation underlying chemoresistance of ACCS-M GFP or CSCs may contribute to radioresistance and the radiosensitizing effect of *Brachyury* knockdown. In radiation biology, cells in the late S-phase are especially resistant, and cells in the G2/M-phase are the most sensitive, to ionizing radiation (45). CSCs in the breast cancer cell line MDA-MB231 are shifted to the S- and G2-phases and are radioresistant. Cyclin D and E protein levels are consistent with this profile, suggesting the involvement of homologous recombination repair in the radioresistant phenotype (46). Therefore, cell cycle regulation in ACCS-M GFP cells may be a key factor underlying radioresistance. This mechanism is an important area of future investigation.

Clinically, CRT and multidrug chemotherapy reduce cancer cell viability by complementarily targeting cellular vulnerabilities. However, CSCs survive these treatments, because they do not target CSC cell cycle regulation. As shown in Fig. 8, *Brachyury* knockdown significantly enhanced the effect of CRT *in vitro* for all tested anticancer drugs to which cells were resistant as a single drug. These data support the conclusion from our previous study that *Brachyury* knockdown forcibly differentiates CSCs, causing them to lose their sternness. Furthermore, the effect of *Brachyury* knockdown was significantly stronger than that of *SOX2,* a conventional stem cell regulatory gene. Multiple regulatory genes are believed to regulate cell sternness. However, we have shown that knockdown of a single gene, *Brachyury,* silenced multiple regulatory genes simultaneously. Hence, *Brachyury* knockdown may be an important therapeutic approach and should be further investigated for clinical use.

In conclusion, this study presents evidence that *Brachyury* knockdown reduces the invasiveness and chemo- and radio-resistance of CSCs *in vivo* and suggests that *Brachyury* knockdown is a useful therapeutic tool for sensitizing CSCs to conventional chemoradiotherapy.

## Figures and Tables

**Figure 5.**
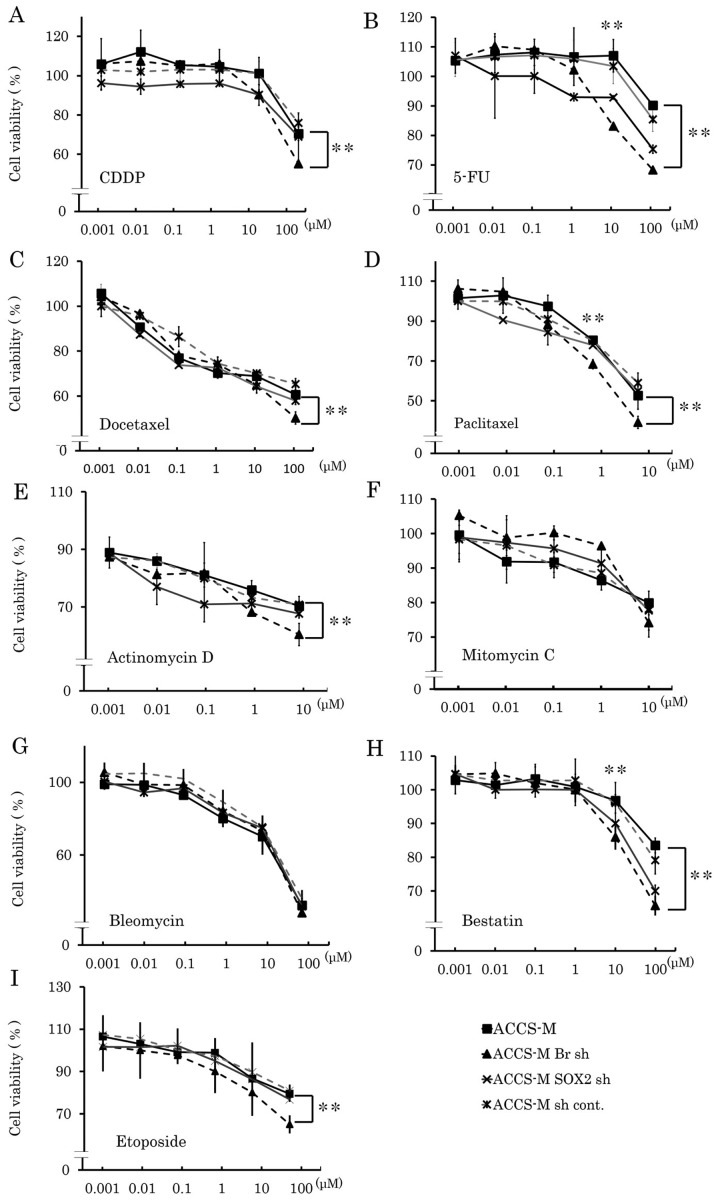
Effect of *Brachyury* and *SOX2* knockdown on chemosensitivity of ACCS-M GFP cells (A–I). Cell viability after application of various types and concentrations of anticancer drugs to ACCS-M GFP, ACCS-M sh cont., GFP ACCS-M shBr GFP, and ACCS-M shSOX2 GFP cells. Error bars indicate standard deviation. ^**^P<0.05.

**Table I.** Measured IC_50_ (*μ*M) of each anticancer drug.

	IC_50_ (*μ*M)				
Anticancer drug	ACCS	ACCS-M	ACCS-M sh cont.	Br sh	SOX2 sh
Cisplatin	353.4	527.6	529.1	360.2	403.4
5-Fluorouracil	463.5	842.9	835.1	623.7	673.6
Docetaxel	0.9	320.5	315.8	104.5	248.5
Paclitaxel	0.75	17.3	18.5	6.7	18.8
Actinomycin D	3.9	43.6	42.1	27.5	35.5
Mitomycin C	33.8	49.8	47.1	40.2	48.2
Bleomycin	41.1	57.8	58.6	50.2	55.8
Bestatin	459	558.8	549.3	306.7	325
Etoposide	N.D.	N.D.	N.D.	N.D.	N.D.

N.D., not determined.
